# Assessment of Renal Function Based on Dynamic Scintigraphy Parameters in the Diagnosis of Obstructive Uro/Nephropathy

**DOI:** 10.3390/jcm10030529

**Published:** 2021-02-02

**Authors:** Paweł Cichocki, Krzysztof Filipczak, Zbigniew Adamczewski, Jacek Kuśmierek, Anna Płachcińska

**Affiliations:** 1 Department of Nuclear Medicine, Medical University of Lodz, 92-216 Łódź, Poland; pawel.cichocki@umed.lodz.pl (P.C.); jacek.kusmierek@umed.lodz.pl (J.K.); 2 Department of Quality Control and Radiation Protection, Medical University of Lodz, 92-216 Łódź, Poland; krzysztof.filipczak@umed.lodz.pl (K.F.); anna.plachcinska@umed.lodz.pl (A.P.)

**Keywords:** dynamic renal scintigraphy, split function, uropathy, nephropathy, technetium Tc 99m-ethylenedicysteine

## Abstract

This study evaluates the usefulness of parameters allowing assessment of renal function in absolute values in dynamic renal scintigraphy (DRS) with ^99m^Tc-ethylenedicysteine (^99m^Tc-EC) uptake constant (K), mean transit time (MTT), and parenchymal transit time (PTT) in the diagnosis of obstructive uro/nephropathy. The study included 226 people: 20 healthy volunteers, for whom normative values of assessed parameters were determined, and 206 patients. Reproducibility of results obtained by two independent operators, specificity, correlation with estimated GFR (eGFR), and Cohen’s kappa were used to evaluate reliability of assessed parameters. Normative values were as follows: K ≥ 1.6, MTT ≤ 250 s, and PTT ≤ 225 s. Reproducibility of determination of K (r_s_ = 0.99) and MTT (r_s_ = 0.98) was significantly higher than that of PTT (r_s_ = 0.95) (*p* = 0.001). Specificity was 100% for K, 81% for MTT, and 91% for PTT. Correlation of eGFR with K (r_s_ = 0.89) was significantly higher than with PTT (r_s_ = 0.53) and with split function (SF) (r_s_ = 0.66) (*p* < 0.0001). Cohen’s kappa was κ = 0.89 for K, κ = 0.88 for MTT, and κ = 0.77 for PTT. In a group of patients where standard DRS parameters are unreliable (bilateral obstructive uro/nephropathy or single functioning kidney), the use of K (the most effective among assessed parameters) changed the classification of 23/79 kidneys (29%). K enables reproducible assessment of absolute, individual kidney function without modifying routine DRS protocol. Diagnostic value of MTT and PTT is limited.

## 1. Introduction

One of the primary indications for dynamic renal scintigraphy (DRS) is the diagnosis of obstructive uro/nephropathy. Typically, this examination is assessed qualitatively (visually) and quantitatively, using parameters such as differential renal function (split function—SF), time of the peak of renographic curve (T_MAX_), and time of renographic curve reaching to half the peak value (T_1/2_) [1]. However, SF only assesses relative function of each kidney, while the reliability of time parameters is limited and depends, for example, on the method and speed of radiopharmaceutical administration or on the patient’s hydration level.

For this reason, it is necessary to study additional, reliable, and reproducible quantitative parameters that would allow the assessment and monitoring of renal function in absolute values.

Processing units of modern gamma cameras usually have software allowing determination of ^99m^Tc-diethylenetriaminepentaacetic acid (^99m^Tc-DTPA) clearance for the determination of glomerular filtration rate (GFR) or ^99m^Tc-mercaptoacetyltriglycine (^99m^Tc-MAG_3_) clearance for the determination of tubular extraction rate (TER) based on DRS study (camera-based clearance). These procedures were developed by Schlegel et al. for ^131^I-orthoidohippurate (^131^I-OIH) [2] and by Gates for ^99m^Tc-DTPA [3] and were later adapted also for ^99m^Tc-MAG_3_. These methods are more accessible than the most accurate and precise radioisotope technique for determining GFR based on a multisample clearance of ^99m^Tc-DTPA. However, their accuracy is being questioned and they require taking into account additional information, such as precise measurement of the activity of radiopharmaceutical administered to the patient or measuring depth of kidneys in order to correct the attenuation of radiation by tissues located between the camera detector and the kidney [4]. Perhaps this is why camera-based clearance methods have not yet found wider application in routine diagnostics. Protocol for the calculation of ^99m^Tc-ethylenedicysteine (^99m^Tc-EC) camera-based clearance was not developed yet.

The aim of this study was to assess diagnostic potential of three different parameters allowing the assessment of renal function in DRS in absolute values, generated using an original software developed in our Department. These are as follows: an original parameter-uptake constant (K), proportional to the clearance function of a kidney, as well as transit time of radiopharmaceutical through the whole kidney (mean transit time—MTT) and its cortex (parenchymal transit time—PTT).

## 2. Experimental Section

### 2.1. Material

The study included a total of 226 people aged 18–84 years (mean: 53), divided into two groups.

Group I—normative, consisted of 20 healthy volunteers (40 kidneys). In this group, ultrasound of the urinary system was performed and serum levels of urea and creatinine were determined on the same day as the DRS study. The criteria for inclusion into group I were as follows: no history of previous or ongoing urinary system diseases or other diseases that could lead to impaired renal function (such as systemic lupus, diabetes, or uncontrolled hypertension); the ultrasound examination of the kidneys showed no features of congenital anomalies, urolithiasis, hydronephrosis, scars or other focal lesions (including cysts), and the results of biochemical tests (serum urea and creatinine levels as well as estimated GFR (eGFR) calculated from Chronic Kidney Disease Epidemiology Collaboration (CKD-EPI) formula) were within normal range.

Group II consisted of 206 adult patients, who were routinely examined at our Department in years 2015–2019, selected retrospectively based on archived scintigraphic data. Patients with uncontrolled diabetes or hypertension and patients with suspicion of renovascular hypertension referred for DRS with Captopril test were excluded from the study. This group included 3 subgroups:

- IIA—62 patients (124 kidneys) with low probability of obstructive uro/nephropathy, whose renal function was assessed as normal based on routine DRS evaluation
- IIB—92 patients (173 kidneys; some patients had only one functioning kidney), whose medical records had up-to-date serum creatinine results (measured no more than 2 weeks before DRS)
- IIC—61 patients (79 kidneys) with no or only trace function of one of the kidneys (SF < 5%) or bilateral obstructive uropathy, where SF values are unreliable (Table 1).

### 2.2. DRS Protocol

All volunteers and patients underwent DRS performed according to the routine protocol used in our Department. All subjects drank 0.5 L of water approximately 30 min before the examination and urinated just before radiopharmaceutical injection [1]. DRS was performed in supine position with GE Healthcare scintillation cameras: Infinia Hawkeye 1, Infinia Hawkeye 4 or Optima NM/CT 640, equipped with low-energy general purpose collimators (LEGP), after administration of standard activity of 111 MBq ^99m^Tc-EC. Field of view covered both the kidneys and the heart. Image acquisition was performed in posterior view, using 128 × 128 matrix. In patients whose renographic curves in the base study remained above 30% of the peak value up to the 20th minute of the study, DRS was extended by a diuretic test performed according to the “F + 20” protocol and image acquisition was continued for additional 10 min [5].

Initial evaluation of all studies, performed by the same nuclear medicine specialist, consisted of visual assessment of sequential, 2 min scintigraphic images and analysis of standard quantitative parameters—SF, T_MAX_, and T_1/2_. In group II, archived medical records were also analyzed, and patients with current serum creatinine level results were qualified to subgroup IIB.

Due to the retrospective character of the study and sometimes incomplete clinical data in archived documentation, further classification of kidneys was based only on the standard evaluation of scintigraphic data. Kidneys with features of obstructive uropathy or nephropathy were distinguished based on the following criteria. No decrease of the renographic curve (“cumulative curve”) or its decrease by less than 50% of the peak value after the diuretic test, in the absence of the features of obstructive nephropathy described below, was classified as a feature of obstructive uropathy. Kidneys meeting at least two of the following three criteria were classified as nephropathic: SF < 42%, T_MAX_ > 6 min, and presence of uptake defects in the peripheral part of the kidney in the visual assessment of sequential images of uptake phase (between 2nd and 3rd minute of the study), performed by two experienced physicians (determined by consensus). At least two criteria are required, because one criterion can be present in normal kidneys (e.g., a small, but properly functioning kidney can have SF < 42%). On the basis of these criteria, patients were classified into subgroups IIA and IIC (independent from subgroup IIB).

### 2.3. Additional Post-Processing

Further analysis of scintigraphic data (post-processing) of each volunteer and patient was carried out by the same nuclear medicine specialist with an open-source program—ImageJ—using an original plug-in developed in our Department. The program automatically summed scintigraphic images in the posterior view from the base DRS study (without the diuretic test) to 60 20 s images. Each of the images was then smoothed twice with a 3 × 3 mask linear filter [6].

**Figure 1:**
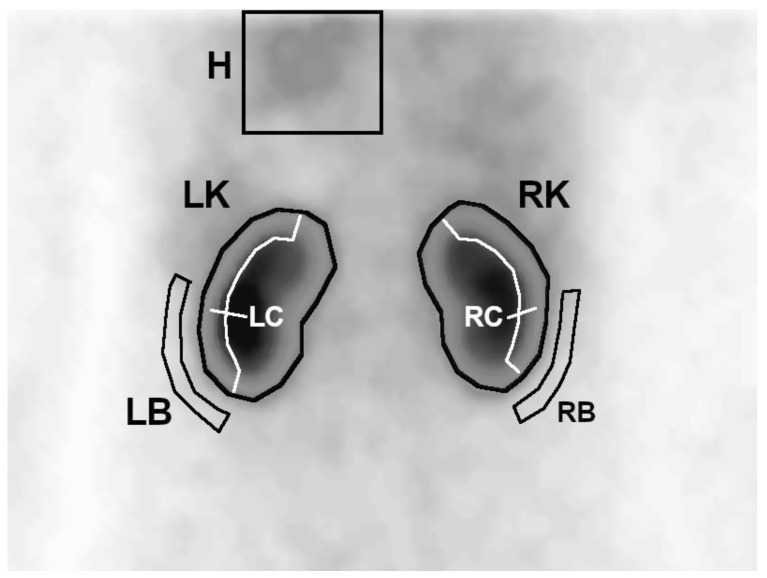
Generation of regions of interest (ROIs) of the heart (H), kidneys (LK and RK), background (LB and RB) and kidney cortex (LC and RC).

On the first summed image, a region of interest (ROI) covering the left ventricle with a large margin of background was determined. Then, on summed images from the uptake phase, whole kidney ROIs were generated semiautomatically—the program plotted an area with activity higher than the threshold value, which was set manually so that the contoured area covered the whole kidney. In the next step, 3-pixel-wide ROIs of the cortical layer (parenchyma) and background were automatically generated for each of the kidneys, based on the previously plotted whole kidney ROIs (Figure 1).

Due to poor visibility of some small and/or nephropathic kidneys on 20 s images, in some cases it was necessary to plot the whole kidney ROI by hand and manually correct automatically determined parenchymal and background ROIs.

These ROIs were then used to generate time–activity curves showing the concentration of the radiopharmaceutical in the heart *H*(*t*), kidneys *R*(*t*), and their parenchyma *Rc*(*t*). The cardiac curve was generated from 20 pixels, with the highest activity selected automatically from the large heart ROI. Remaining curves were determined as a sum of counts in a given ROI to time.

This post-processing was performed again, using the same procedure, by another, independent specialist for patients from subgroup IIB.

Uptake constant K was determined based on the Rutland space [6,7]. It is a space described by the ratio of variables forming the renal and cardiac curves. In the first phase of DRS examination (uptake phase—between 2nd and 3rd minutes), the distribution of points in this space is approximated by a straight line described by the following equation:

(1) $$\frac{R\left(t\right)}{H\left(t\right)}=K\cdot \frac{\int_{0}^{t}H\left(t\right)dt}{H\left(t\right)}+F$$

where *R*(*t*)—background-corrected renographic curve, *H*(*t*)—background-corrected cardiac curve, *F*—blood background subtraction factor, and *K*—uptake constant (fraction of blood activity taken up by the kidney per time unit).

According to Rutland’s theory, *K* represents clearance function of the kidney, although only in proportional values, hence it is a unitless parameter.

Renal *R*(*t*), parenchymal *Rc*(*t*), and cardiac *H*(*t*) curves were used for deconvolution analysis. Each of the *R*(*t*) and *Rc*(*t*) curves were corrected by subtracting the background activity (calculated as mean counts in the background ROI multiplied by the size of kidney ROI). MTT and PTT were determined from the curves plotted in this way, using matrix deconvolution technique.

### 2.4. Statistical Analysis

The data were described by means of simple descriptive statistics of location and dispersion (mean, median, percentage, range, and standard deviation). Normality of distributions was verified by Shapiro–Wilk’s test. Normative values for analyzed parameters with confirmed normal distributions were assessed as mean ± 2 or 3 standard deviations in the group of healthy adults. Distributions of selected quantitative parameters, some of which were not normally distributed, were compared between different groups by nonparametric Mann–Whitney U test.

Diagnostic performance of parameters of interest was evaluated by sensitivity, specificity, and inter-observer reproducibility. Relations between parameters of interest were assessed using nonparametric Spearman’s rank correlation coefficient and coefficient of determination r^2^. Agreement of results was analyzed using Cohen’s kappa coefficient.

In all analyses, statistical significance was considered achieved for a value of *p* ≤ 0.05.

Calculations were performed by means of Statistica v13.1 and LibreOffice v6.3 software.

### 2.5. Analysis of Generated DRS Parameters

Parameters K, MTT, and PTT in subgroup IIB (*n* = 173 kidneys) were determined by two independent operators. Correlation of results obtained by both operators was used as a measure of reproducibility of determination of each parameter. The agreement of results obtained by two independent operators (values within normal limit vs. values below or above norm) was also assessed.

In order to assess reliability of normative values of assessed parameters, determined in the group of healthy volunteers, their specificity was determined in a group of patients without features of obstructive uro/nephropathy in routinely evaluated DRS (IIA). Differences between mean values of assessed parameters in the above-mentioned groups were also analyzed.

Next, in group IIB, correlation of K, PTT, and SF with estimated GFR of each kidney (single-kidney eGFR—SKeGFR) was assessed. eGFR was calculated based on the current serum creatinine level using CKD-EPI formula, recommended by the guidelines of the international work group—Kidney Disease: Improving Global Outcomes (KDIGO) [8]. Then, its value was multiplied by the differential renal function of each kidney in DRS.

MTT was not compared with SKeGFR, because its value is influenced by both renal uptake function and the efficiency of urine outflow to the bladder. Instead, diagnostic efficacy of MTT in the diagnosis of obstructive uropathy was characterized by basic parameters of the diagnostic test—sensitivity and specificity, with diuretic test used as a reference method.

In a group of patients with single functioning kidney or features of bilateral uropathy (IIC), a comparative differential analysis of obstructive uro/nephropathy was performed based on standard and assessed DRS parameters.

## 3. Results

### 3.1. Reproducibility and Normative Values

In order to assess reproducibility of K, MTT, and PTT, susceptibility of the method of their determination to errors resulting from the subjective factor was analyzed. Scintigraphic data of patients from subgroup IIB (*n* = 173 kidneys) were analyzed by two independent operators. Correlation coefficients and r^2^, used as measures of the reproducibility of determination of assessed parameters, were as follows: for K, r_s_ = 0.99 (r^2^ = 0.98), for MTT, r_s_ = 0.98 (r^2^ = 0.96), and for PTT, r_s_ = 0.95 (r^2^ = 0.90) (*p* < 0.000001). Correlations of K and MTT were significantly stronger than those of PTT (*p* = 0.001).

In group I—control, SF was within the range of 42–58%, T_MAX_ was no longer than 6 min, and T_1/2_ did not exceed 17 min. Distributions of values of assessed parameters in this group were normal. Mean – 2 standard deviations for K and + 3 SD for MTT and PTT were considered normative values.

These normative values were used to determine specificity of assessed parameters in an independent group IIA—patients with low probability of obstructive uro/nephropathy and a normal result of routinely analyzed DRS. In this group, K was within normal range in all kidneys and its mean value did not differ significantly from the results of healthy volunteers. MTT was normal in 100/124 (81%) of the kidneys and PTT in 113/124 (91%) of cases. (Table 2).

Based on these normative limits, results obtained by each operator in subgroup IIB were categorized as within normal range or below/above norm. The agreement of the results is presented in Table 3.

**Figure 2:**
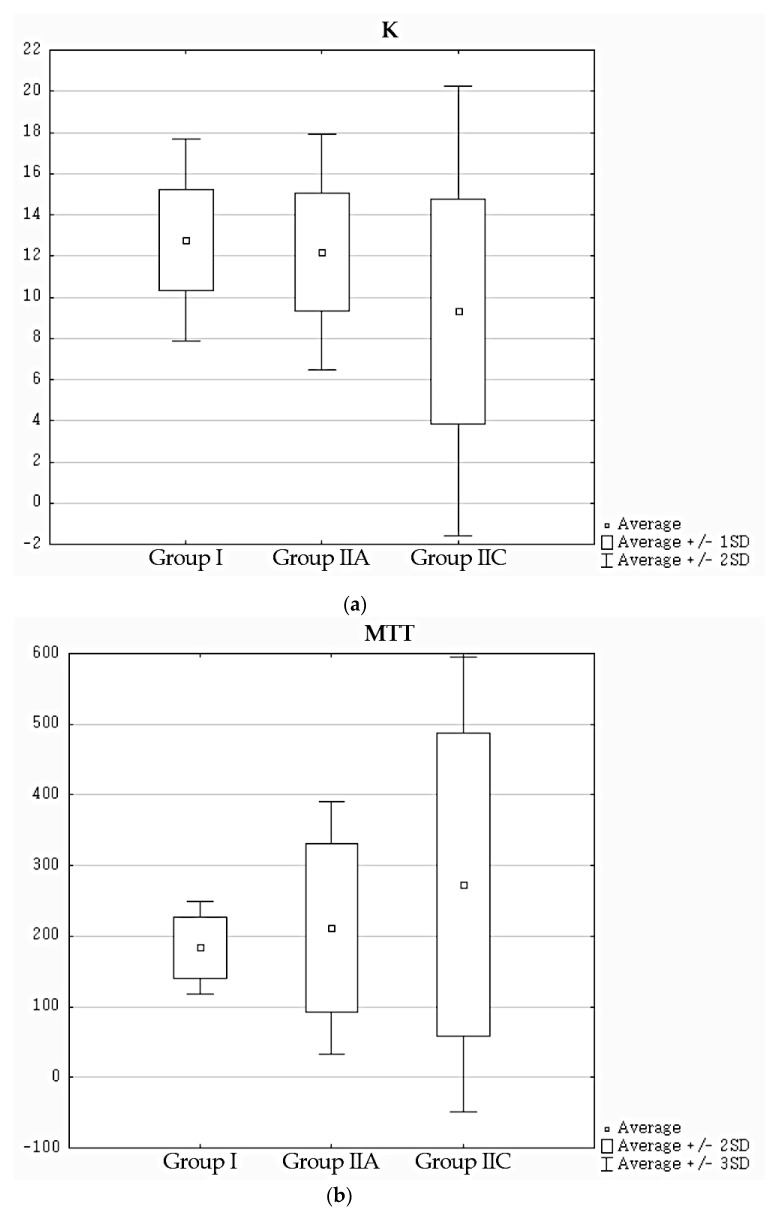

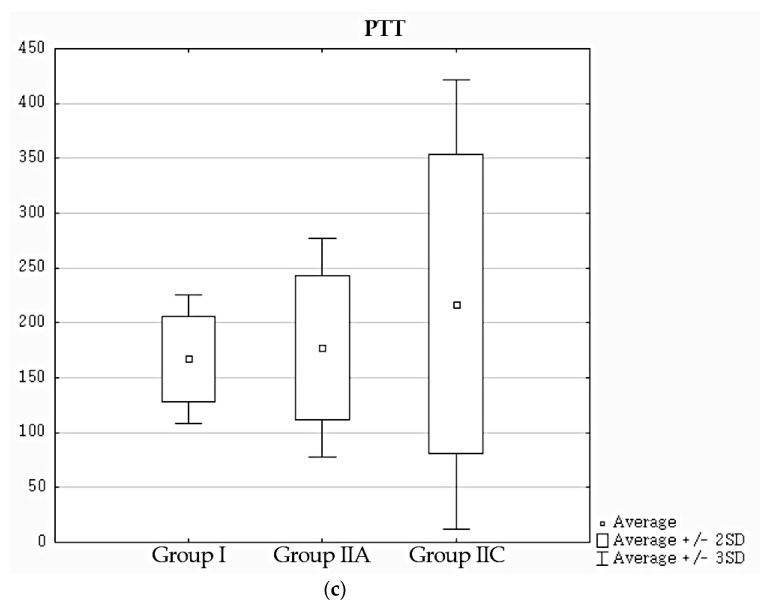
Distribution of values of (**a**) K, (**b**) MTT, and (**c**) PTT in selected groups.

The distribution of values of assessed parameters in groups I, IIA, and IIC is presented in Figure 2. There were no statistically significant differences between mean values of K between groups I and IIA (*p* = 0.38), while in case of MTT and PTT, the differences were at the edge of statistical significance (*p* = 0.05 and *p* = 0.07, respectively). Differences in mean values between groups I and IIC were statistically significant for all assessed parameters—in case of K they were lower (*p* = 0.0002), while in case of MTT and PTT they were higher (*p* < 0.00001 in both cases). Group IIB was not included in this comparison, since it includes a wide range of patients both with normal and impaired kidney function.

### 3.2. Correlation with eGFR

**Figure 3:**
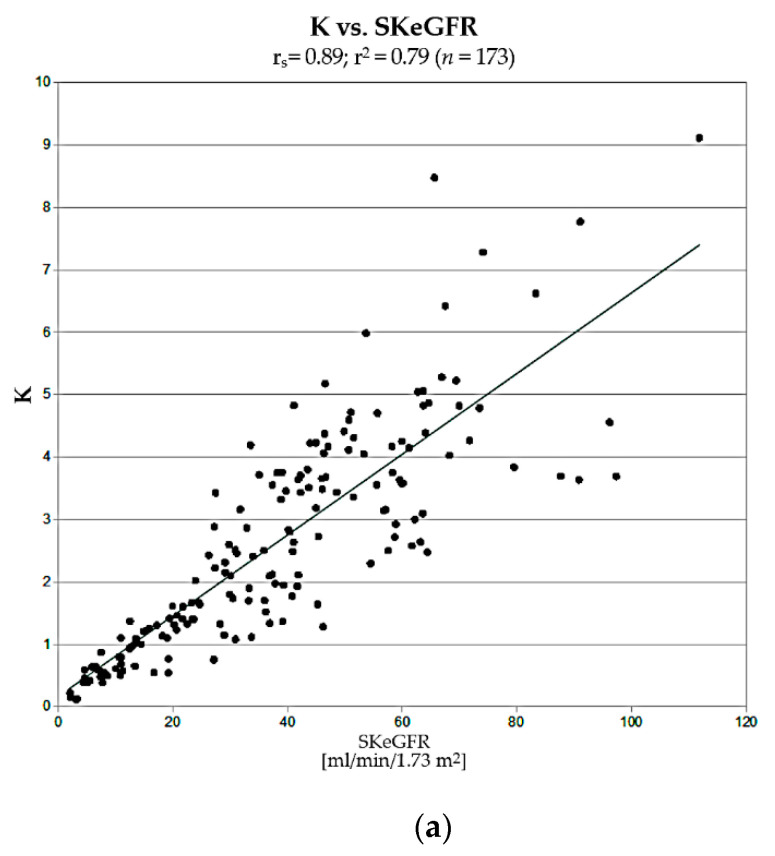

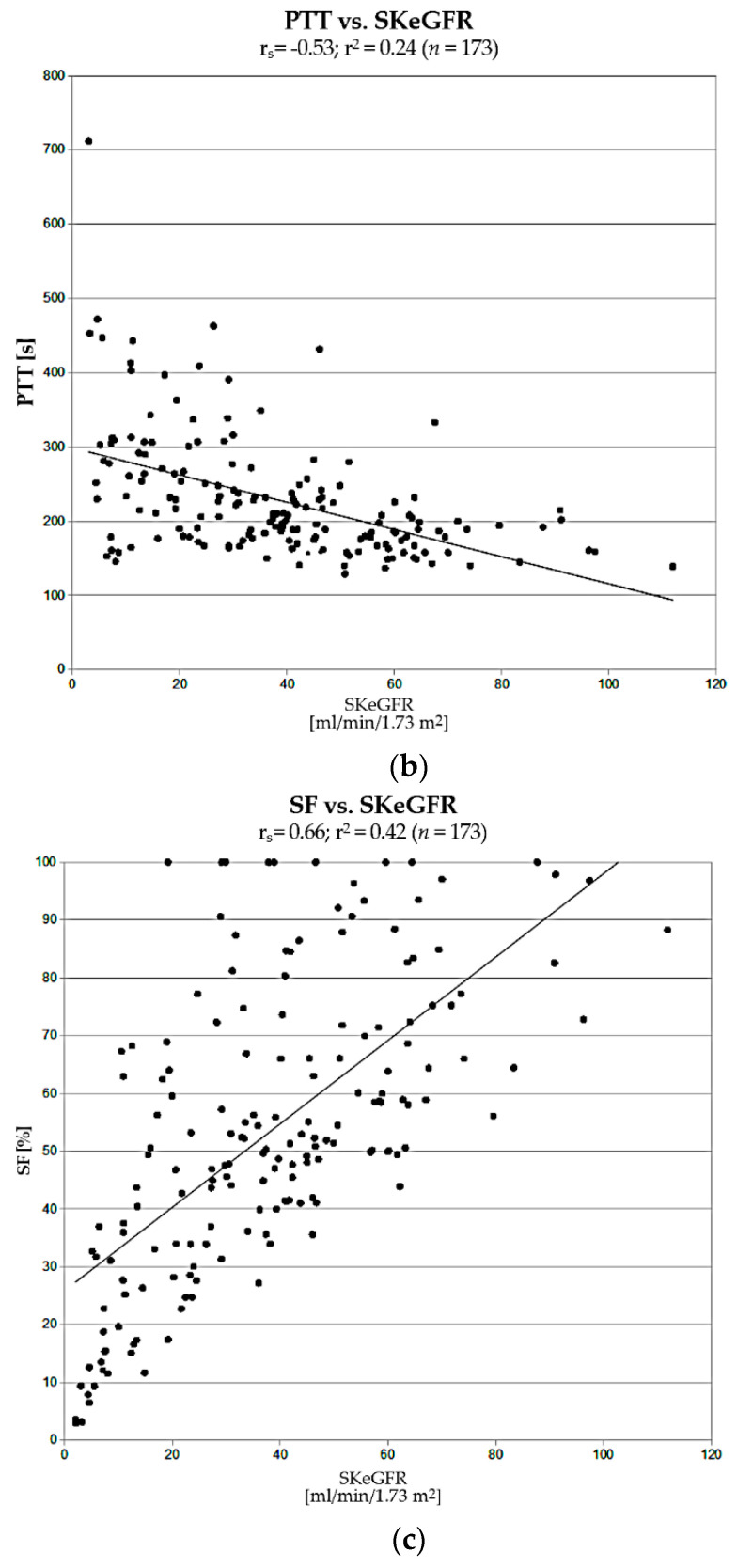
Correlations of single-kidney estimated GFR (SKeGFR) with (**a**) K, (**b**) PTT, and (**c**) split function (SF).

Values of K, PTT, and SF showed a significant correlation with SKeGFR in group IIB, r_s_ = 0.89, r^2^ = 0.79 (*p* < 0.000001), r_s_ = −0.53, r^2^ = 0.24 (*p* < 0.000001), and r_s_ = 0.66, r^2^ = 0.42 (*p* < 0.000001), respectively. These correlations are presented in Figure 3.

Although all correlations were statistically significant, correlation of uptake constant K with SKeGFR and its correlation coefficient r^2^ were significantly stronger than in the case of PTT (*p* < 0.0001) and SF (*p* < 0.0001).

### 3.3. Preliminary Assessment of Clinical Utility of K

Since the reliability of K was considered to be higher than that of PTT (due to better reproducibility with significantly higher agreement of results between operators, higher specificity, and stronger correlation with SKeGFR), clinical utility of this parameter in differential diagnosis of obstructive uro/nephropathy was preliminarily assessed.

**Figure 4:**
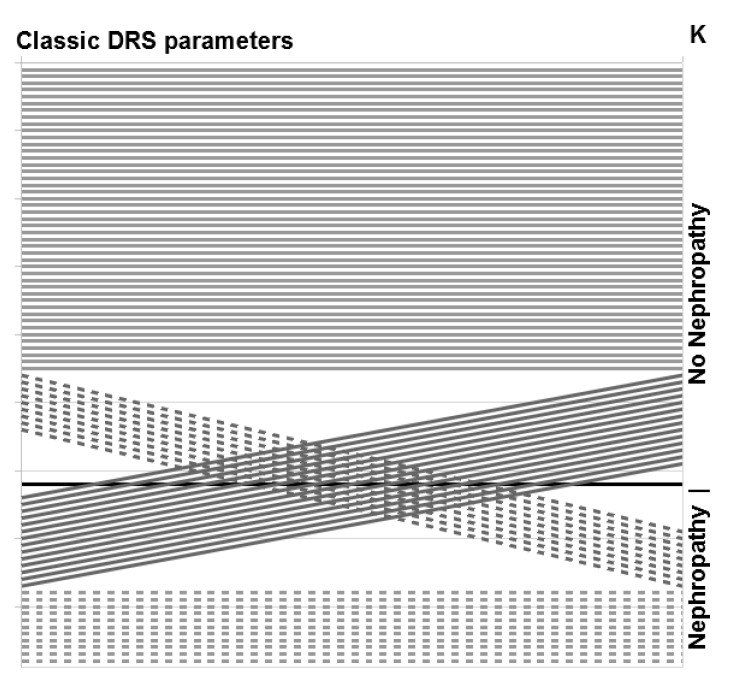
Change of qualification of kidneys from routine dynamic renal scintigrapy (DRS) evaluation in group IIC based on K value.

Based on standard DRS criteria, described in methodology, in group IIC, no features of obstructive uro/nephropathy were found in 36 kidneys, obstructive uropathy was diagnosed in 18 kidneys, while features of obstructive nephropathy were found in 25 kidneys. Application of K changed the qualification of 23 out of 79 kidneys (29%). In 14 cases (18%), it changed the classification from nephropathy to uropathy, while in 9 cases (11%), K value suggested nephropathy with no such features found in routine DRS evaluation (Figure 4).

### 3.4. Assessment of Clinical Utility of MTT

Out of 362 analyzed kidneys from group II, in 108 a cumulative curve was observed in base DRS study, which in 16 cases decreased below 50% of peak value after diuretic test. The remaining 92 cases showed features of obstructive uropathy.

MTT values in group II ranged from 131 s to 639 s. Sensitivity of this parameter in the diagnosis of obstructive uropathy, in relation to the reference diuretic test, was 77% (71/92 kidneys), while its specificity was 76% (204/270 kidneys).

**Figure 5:**
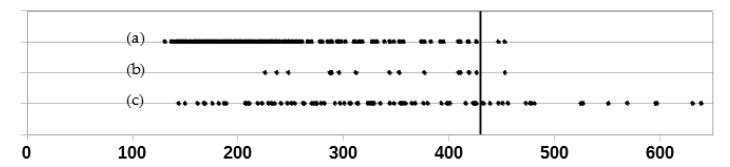
Distribution of MTT values in group II: (**a**) all kidneys without features of obstructive uropathy, (**b**) kidneys without features of obstructive uropathy, with cumulative renographic curve in base study, (**c**) all kidneys with features of obstructive uropathy. Vertical line marks MTT value of 430 s.

Among all kidneys without features of obstructive uropathy, in 268 out of 270 (99%) MTT was below 430 s (in remaining 2 kidneys its value was 447 s and 453 s). In the group of 16 kidneys with a cumulative curve in base study, but without obstructive uropathy, its value in 15 cases (94%) also did not exceed 430 s (except for one of the above-mentioned kidneys with MTT = 453 s). In 92 kidneys with features of obstructive uropathy, MTT was above 430 s in 20 cases (22% of the kidneys). Distribution of the MTT values is shown in Figure 5.

## 4. Discussion

In clinical practice, DRS is the most commonly used radioisotope technique in the diagnosis of urinary system. Despite numerous advantages, however, it has some limitations that can be eliminated by supplementing the standard evaluation of the study with additional parameters that enable an assessment of renal function in absolute values. Techniques that allow such evaluation using only scintigraphic data obtained in DRS, without the need to collect blood or urine samples or perform other additional tests, are especially valuable. Several protocols have been developed to determine renal clearance of radiopharmaceuticals in this way, and some of them have been integrated into software of modern gamma cameras. However, these camera-based techniques require acquisition of additional—nonstandard—data, such as the exact activity of the injected radiopharmaceutical (taking into account activity remaining in the syringe after administration) or measurement of the depth of the kidneys. This requires modifications of the routine DRS protocol and extends and complicates the course of the entire study. Moreover, these protocols have not yet been adapted for DRS performed with ^99m^Tc-EC—the latest radiopharmaceutical introduced to the diagnostics of the urinary system.

Software developed in our Department allows determination of additional quantitative parameters describing kidney function in absolute values—original parameter, uptake constant (K), mean transit time (MTT), and parenchymal transit time (PTT)—without the need of any modifications to the routine DRS protocol.

Our method of calculating K in DRS performed with ^99m^Tc-EC was based on Rutland’s theory [7]. According to its assumptions, the parameter K is proportional to the clearance of ^99m^Tc-EC and thus to the effective renal plasma flow (ERPF) of a given kidney. In 1985 Rehling et al. proposed a method, similar to ours, of determining iptake index (UI)—a parameter proportional to ^99m^TcDTPA clearance (thus GFR of a given kidney). Rehling et al. proved that UI correlates very closely with GFR value (determined with the radioisotope method based on the multisample clearance of ^99m^TcDTPA)—r_s_ = 0.97 [9].

It should be emphasized that in the Equation (1), which is the basis of this method, cardiac curve—H(*t*)—appears three times, so it has a decisive influence on the value of K (which is the slope of the function described by this equation). In his work, Rehilng also emphasizes the important role of the vascular background chosen to generate the cardiac curve, necessary to calculate the UI. Therefore, susceptibility of the method of generating this curve to errors resulting from the subjective factor should be minimized. For this purpose, in our work cardiac curve was generated from the sum of counts in 20 pixels, with the highest values in the large ROI covering the area of the heart. Additionally, background-correction of renographic curves was applied in this study, using automatically generated background ROIs.

This study also assessed the diagnostic value of MTT and PTT, other parameters supplementing routine DRS examination, after optimization of the methodology of their determination. These parameters are assumed to quantitatively represent the transport function of the kidney, based on the transit times of the radiopharmaceutical through the whole kidney and its parenchyma. However, methods of their determination are prone to errors [10,11,12]. In this study, MTT and PTT were determined with matrix deconvolution technique. There are several alternative methods for determining these parameters described in literature [13,14,15,16,17], but there is no clear consensus in regards to their diagnostic usefulness. Apart from positive assessments of the importance of these parameters, there are a number of reports questioning their usefulness [12,18,19,20].

Results of our earlier research showed that methodology of MTT and PTT determination, based on the matrix deconvolution technique, used previously in our Department, already generated reproducible results of MTT. However, reproducibility of PTT determination by different operators (inter-observer reproducibility) was significantly lower [21].

Automation of ROI plotting and automatic selection of pixels used to generate the cardiac curve, used in the current study, limited the influence of the subjective factor on the determination of both PTT and MTT as well as K practically exclusively to the manual selection of the threshold for semiautomatic plotting of whole kidney ROIs. As a result of such automation, we achieved very high reproducibility of determination of assessed parameters, which was for K, r_s_ = 0.99, for MTT, r_s_ = 0.98, and for PTT, r_s_ = 0.95. Agreement of results (both values within normal range or below/above norm) obtained by independent operators was also almost perfect for K (κ = 0.89) and MTT (κ = 0.88), and strong for PTT (κ = 0.77).

Due to high variability of MTT and PTT, some authors recommend setting the limits of their normative values as mean + 3 standard deviations (considering values between 2 and 3 SD as unequivocal) [10]. Following this approach, mean + 3 SD values were chosen as normative values of these parameters in our work, while mean − 2 SD was used for K.

Specificity, assessed in an independent group of patients with no abnormalities in standard DRS evaluation, was used as a measure of reliability of normative values of assessed parameters. K achieved the highest specificity (100%). A slightly lower specificity of PTT (91%) may result from cases where an automatically determined cortical ROI covered fragments of calices. K is determined from the first minutes of the study, before the radiopharmaceutical reaches the pelvicalyceal system, so the above limitation does not apply to it. MTT showed a clear tendency to generate false-positive results (specificity 81%).

Moreover, it should be noted that mean values of K in the normative group I and in the group of patients with normal DRS results (IIA) did not show statistically significant differences. However, in cases of MTT and PTT, the differences between mean values in these groups were at the edge of statistical significance.

The next step in the assessment of usefulness of K and PTT was a comparison of correlations of their values with single-kidney estimated GFR (SKeGFR), which describes renal efficiency, determined for each kidney using CKD-EPI formula (taking into account the percentage of differential renal function). Obtained values of K strongly correlated with SKeGFR (r_s_ = 0.89; r^2^ = 0.79). This correlation was significantly stronger than in cases of PTT vs. SKeGFR and SF vs. SKeGFR.

The fact that the correlation of K vs. SKeGFR is not as close as in the case of UI determined by Rehling (r_s_ = 0.97) is most likely the result of the limitations of our reference method, chosen due to the retrospective nature of our research (only estimated values of GFR were calculated) and slightly different renal biokinetics of ^99m^Tc-EC compared to ^99m^Tc-DTPA. Despite the above limitations, the correlation we obtained was satisfactorily strong, which confirms the validity of the main assumptions of this method.

Measuring the depth of kidneys, for the purpose of attenuation correction, is recommended only in exceptional situations [4,22]. For this reason, in our studies, similarly to Rehling et. al., we did not take these factors into account. Omission of this stage enables determination of K in DRS carried out according to a routine protocol (without any additional measurements or modifications, provided that the heart is in the field of view of the camera), so it is possible to calculate its value a posteriori, only in selected cases, for example, when evaluation of standard DRS parameters results in an unequivocal diagnosis. It also makes it possible to determine this parameter in retrospective studies. This is not possible, for example, in case of camera-based clearance methods according to Gates’ and Schlegel’s protocols.

Considering good correlation of K with SKeGFR, there are theoretical premises supporting its validity for diagnosis of nephropathy. Due to its absolute character, it can be particularly useful in the diagnosis of nephropathy in patients with bilateral impairment of renal function or with a single functioning kidney, in whom standard DRS parameters, SF in particular, may be unreliable. The significant number of cases where application of K changed the classification of a kidney—over 1/4 of the assessed kidneys in our material, shows its considerable potential utility in routine clinical practice, although whether this new classification is correct needs to be further evaluated, due to limitations of the current study.

In our material, there were statistically significant differences between distributions of age in control group I and retrospectively selected group II. This difference can be explained by the fact that the incidence of diseases potentially affecting renal function, that excluded candidates from group I, increases with age. However, there was no statistically significant difference between group I and patients from subgroup IIA, where specificity of assessed parameters was evaluated.

This preliminary assessment gives grounds for undertaking further long-term, thorough, prospective clinical studies, also taking into account all comorbidities potentially affecting kidney function. This will allow to finish validation of this parameter for use in differential diagnosis of obstructive uro/nephropathy.

PTT achieved lower specificity, and its correlation with renal efficiency measured by SKeGFR was significantly weaker than in case of K. Moreover, its determination requires generation of additional ROIs of renal parenchyma (cortical layer), which may be a source of potential errors. Hence, the reproducibility of its determination by two independent operators and agreement between obtained results, although high, were nevertheless lower than in cases of K and MTT, even despite almost complete automation of the ROI plotting process. The results of the research carried out in this study clearly indicate that the original uptake constant K is a better and more practical parameter for the assessment of renal function in DRS.

Relatively low specificity of MTT in addition to the long-established and well-documented high diagnostic efficacy of base DRS study with diuretic test in the diagnosis of obstructive uropathy undermines the usefulness of this parameter. In this study, however, it was observed that obstructive uropathy is practically always present when MTT values are exceeding 430 s. This can provide useful information in situations where a diuretic test is risky (e.g., furosemide allergy or low blood pressure), or the test was nondiagnostic (e.g., study interrupted too early due to patient’s need to urinate). In such cases, a cumulative renographic curve in base DRS study combined with MTT value above 430 s allows for the diagnosis of obstructive uropathy with about 95% probability, even without the diuretic test.

It is worth noting that all studies carried out in this paper concern adults. The topic to be considered in future research is the application of K parameter in children, after determining appropriate normative values. The developed method may also be adapted for DRS performed with ^99m^Tc-MAG3 (to calculate a similar parameter to K, proportional to its clearance).

## 5. Conclusions

1. Original parameter-uptake constant K extends the possibilities of DRS by reproducible, quantitative assessment of the individual function of each kidney in absolute values.
2. Determination of K does not require modification of the routine DRS protocol. Thus, it can be obtained as part of the post-processing of scintigraphic data (after conducting a standard DRS), either routinely or only in the selected clinical situations.
3. PTT has lower diagnostic potential than K, and the methodology of its determination is more complicated, which undermines its usefulness in the diagnosis of obstructive uro/nephropathy.
4. MTT may be useful for detection of obstructive uropathy in the rare circumstances where a diuretic test is contraindicated or its results are unequivocal.

## Figures and Tables

**Table 1:** Summary of demographic data of examined subjects.

	Number of Examined Subjects (Number of Kidneys in Brackets) and Their Sex			Age of Examined Subjects				
	Total	Female	Male	Min.	Max.	Avg. ± SD	Median	Difference with Group I
Group I	20 (40)	15	5	26	66	50 ± 11	51	
Group II	206 (362)	135	71	18	84	53 ± 18	61	*p* = 0.0003
Subgroup IIA	62 (124)	48	14	18	79	48 ± 17	48	*p* = 0.62
Subgroup IIB	92 (173)	57	35	18	84	55 ± 16	59	*p* = 0.0020
Subgroup IIC	61 (79)	38	23	18	83	57 ± 19	61	*p* = 0.0034

**Table 2:** Values of examined parameters (uptake constant K, mean transit time MTT and parenchymal transit time PTT) in group I and their specificity.

Parameter	Mean	SD	Normal Limits	Specificity
K	3.26	0.85	≥1.6	100%
MTT	184	22	≤250 s	81%
PTT	167	20	≤225 s	91%

**Table 3:** Agreement of results obtained by two independent operators (Op1 and Op2) with Cohen’s kappa coefficients.

K	Op1 ≥ 1.6	Op1 < 1.6	MTT	Op1 ≤ 250	Op1 > 250	PTT	Op1 ≤ 225	Op1 > 225
Op2 ≥ 1.6	73	5	Op2 ≤ 250	69	2	Op2 ≤ 225	86	7
Op2 < 1.6	2	50	Op2 > 250	6	53	Op2 > 225	8	29
κ	0.89		κ	0.88		κ	0.7	

## Data Availability

The data presented in this study are available on request from the corresponding author.
